# Supplementary material for: The interactome of intact mitochondria by cross-linking mass spectrometry provides evidence for coexisting respiratory supercomplexes
Source: Mol Cell Proteomics. 2017 Dec 8;17(2):216–32. doi: 10.1074/mcp.RA117.000470 (PMC5795388; doi:10.1074/mcp.RA117.000470)
Supplement: Supplemental Data [file supp_RA117.000470_133922_0_supp_23978_fzffwf.zip › spectra_annotation/mito_DR_spectra_annotation/58-1-3-1-10-1.pdf]

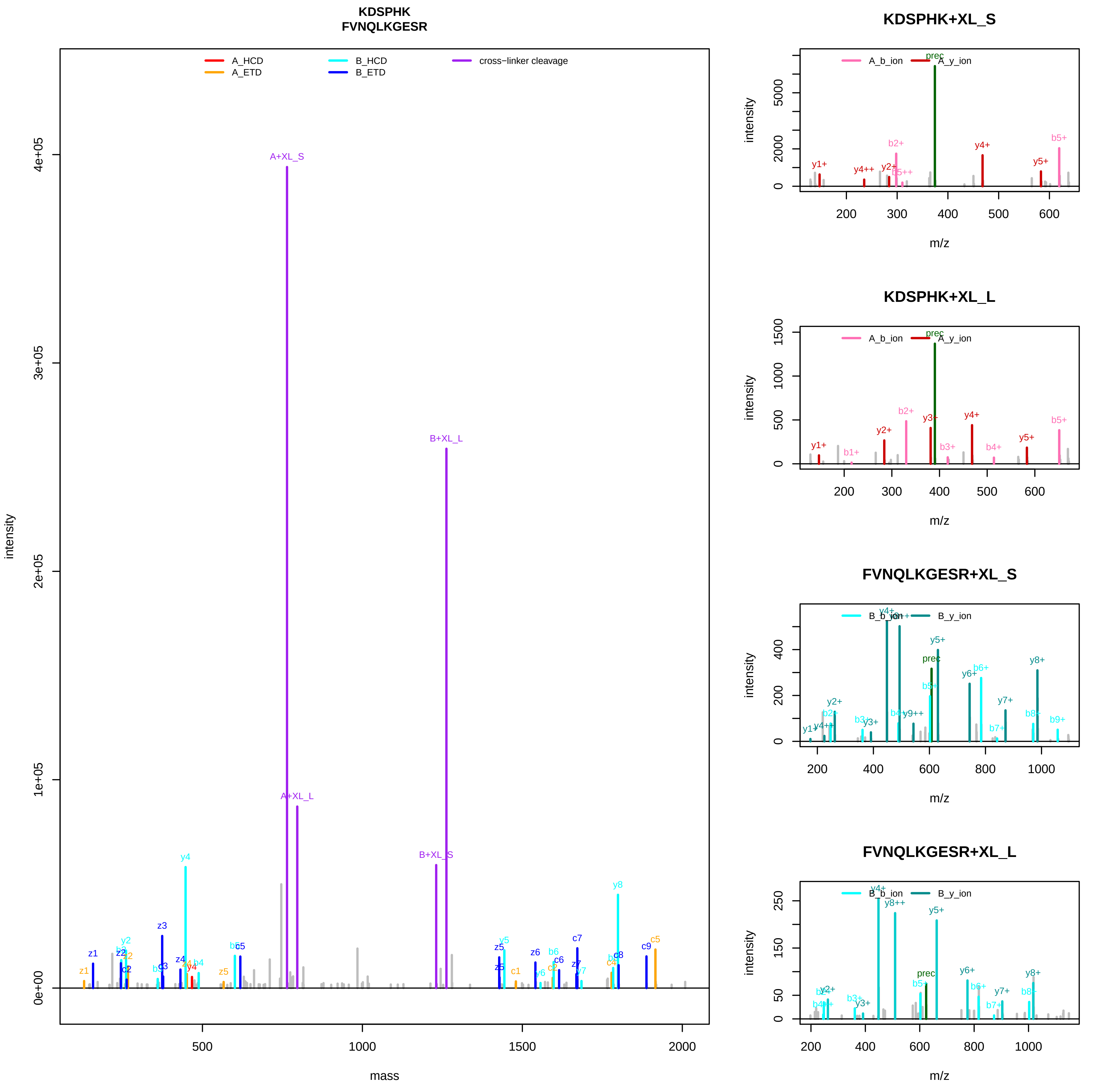
KDSPHK
FVNQLKGESR
A_HCD
A_ETD
B_HCD
B_ETD
cross−linker cleavage
intensity
0e+00
1e+05
2e+05
3e+05
4e+05
A+XL_S
A+XL_L
B+XL_L
B+XL_S
y4
y8
z3
c5
c7
y5
z5
c9
c5
mass
500
1000
1500
2000
KDSPHK+XL_S
A_b_ion
A_y_ion
prec
b2+
y4+
b5+
y1+
y4++
y2+
y5+
intensity
m/z
KDSPHK+XL_L
A_b_ion
A_y_ion
prec
b2+
y3+
y4+
y2+
y1+
b1+
b3+
b4+
y5+
b5+
intensity
m/z
FVNQLKGESR+XL_S
B_b_ion
B_y_ion
y5+
prec
y6+
b6+
y8+
y2+
y7+
b9+
intensity
m/z
FVNQLKGESR+XL_L
B_b_ion
B_y_ion
y4+
y8++
y5+
prec
y6+
y8+
intensity
m/z
